# Medical school origins of award-winning surgeons; analysis of a complete national dataset

**DOI:** 10.1186/s12909-023-04362-6

**Published:** 2023-05-22

**Authors:** S. Steele, G. Andrade, N. Sambandan

**Affiliations:** 1 College of Medicine, Ajman University, University Street, PO Box: 346, Ajman, UAE; 2 Barnet Hospital, Royal Free London NHS Foundation Trust, London, UK

**Keywords:** Medical schools, Medical careers, Award-winners, Globalization, International Medical Graduates

## Abstract

**Background:**

Britain attracts doctors from all over the world to work in the National Health Service. Elucidating the educational backgrounds of award-winning doctors working in the country is potentially an important medical education issue and merit award audit. Using the British clinical merit award schemes as outcome measures, we identify medical school origins of award-winning doctors who have been identified as having achieved national or international prominence.

**Methods:**

The Clinical Excellence Awards/Distinction Awards schemes select doctors in Britain who are classified as high achievers, with categories for national prominence and above. We used this outcome measure in a quantitative observational analysis of the 2019 dataset of all 901 award-winning doctors. Pearson's Chi-Square test was used where appropriate.

**Results:**

Seven medical schools (London university medical schools, Glasgow, Edinburgh, Aberdeen, Oxford, Cambridge and Manchester) accounted for 52.7% of the surgical award-winning doctors in the 2019 round, despite the dataset representing 85 medical schools. Surgeons with the lower grade national awards came from a more diverse educational background of 43 medical schools. International medical graduates accounted for 16.1% of the award-winning surgeons and 9.8% of the award-winning non-surgeons. 87.1% of the surgical award-winners were from European medical schools, whereas 93.2% of the non-surgical award-winners were from European medical schools.

**Conclusions:**

The majority of the award-winning surgeons originated from only seven, overrepresented, medical schools. A greater diversity of medical school origin existed for the lowest grade national merit awards. These comprised 43 medical schools and indicated greater globalization effects in this category. International medical graduates contributed substantially to these award holders; surgical award-winners were more likely to be international medical graduates (16.1%) than non-surgical award-winners (9.8%). This study not only indicates educational centres associated with the production of award-winners but also provides students with a roadmap for rational decision making when selecting medical schools.

## Background

The backbone of any good clinical practice is access to high quality surgical specialists whose intervention is essential for patient management. Britain is unique in having longstanding national merit award systems that reward doctors who are deemed to be performing well. Identifying the educational characteristics of such individuals contributes to our understanding of the pathways most likely to create more of these high-achieving doctors. Our study analyzes the medical school backgrounds of these award-winning surgical clinicians.

In Britain, historically there have been two national merit award schemes in place to reward successful clinicians working in the National Health Service (NHS), the Clinical Excellence Awards Scheme (for Wales and England) and the Distinction Awards Scheme (for Scotland) [1]. Although the Scottish scheme is continuing, the Clinical Excellence Award scheme is currently being iteratively improved and renamed as the National Clinical Impact Awards (NCIA). Applicants self-nominate as part of the initial processes when applying for these awards. The doctors selected for any these awards benefit not only from the positive career and reputational effects but also from the recurring explicitly financial incentives associated with such honours [1].

Although these award schemes were originally established after World War II for the purpose of encouraging senior doctors to support the newly-formed NHS, these awards have been an ongoing subject of discussion in the medical community. Accordingly, the process by which merit awards have been assigned has long been a source of debate/controversy. Consequently, they have been analyzed and discussed with regard to award objectivity [2], distribution by specialty [3] by region [3], by gender [1] by age [4] and by ethnicity [5] but *not by medical school*. Such constructive criticism has led to iterative improvements in the award schemes over the last three decades. Many commentators agree that some system should be in place to reward successful consultants [6] and these awards are viewed as national measures of clinical career success and accounts for their continuing utility more than 60 years after their inception. This original study adds to the educational discussion by relating the surgical and non-surgical award-winners to their medical schools of origin. We place our findings in the context of educational, demographic and career implications for medical students and doctors aspiring to achieve career success [7, 8].

## Methods

The lists of the surgeon award holders and non-surgical specialty award holders were obtained from the Distinction Awards Annual Report, 2020 [9] and the Clinical Excellence Awards Report, 2020 [10] for the 2019–20 round. These lists included both the new awardees and the previous award-winners who continued to hold their awards. The medical schools of origin were identified by using the UK Medical Register [11] and the UK Dental Register [12]. The total number of merit award-winners was 901; the medical school of origin was successfully identified for 99.8% of these clinicians. Consequently, 899 participants were included in the dataset. Award-winning doctors in the publications above, who were designated as specializing in any of the surgical disciplines, were included in this study. In the 2019 award round the following specialties were specified in the databases: surgery, general surgery, otorhinolaryngology/ENT, oral and maxillofacial surgery, dentistry, ophthalmology, cardiothoracic surgery, orthopaedic surgery, plastic surgery, paediatric surgery, urology and obstetrics & gynaecology.

The medical school rankings by the number of merit award-winning alumni were determined by the summation of the number of award-winners of A plus (A^+^), A or B grade (or platinum, gold, silver and bronze award-winners). Only these national level grade Distinction Award and Clinical Excellence Awards were included in this study. When combining the awards from these two systems, A plus award holders were aggregated with platinum award holders, A award holders were aggregated with gold award holders and B award holders were aggregated with silver and bronze award holders.

The rankings of merit award holding medical schools were determined by summation of the number of surgery award holders of A plus (A^+^), A or B grade (or platinum, gold, silver or bronze award holders). Only these national level Clinical Excellence Awards and Distinction Awards were included in this study. When combining the merit awards, A plus award holders were aggregated with platinum award holders, A award holders were aggregated with gold award holders and B award holders were aggregated with silver and bronze award holders.

The rankings of the medical schools by the number of merit award-winning alumni were size corrected by dividing the total number of award holders that were alumni of the medical school by the number of admissions to the undergraduate medical school in the 2019–20 academic year.

The comparison of the distributions of award holders (surgeon merit award-winners versus non-surgeon merit award-winners) was quantified using Pearson's Chi-Square test with the significance level set to *p* < 0.05.

All procedures were performed in compliance with the pertinent guidelines.

Patients and public involvement; no patient involvement.

## Results

There were 224 surgical merit award holders in the 2019 award round and 50.4% of these were either “general surgeons” or non-specific “surgeons”. The surgical specialty that held the most A^+^/platinum awards was obstetrics and gynaecology. The surgical specialty that held the most A/gold awards was dentistry. The surgical specialty that held the most B/silver/bronze awards was surgery/general surgery.

Table 1 shows the ten medical schools whose graduates attained the greatest number of merit awards; these award holders had attained A + (A plus), A, B, platinum, gold, silver or bronze awards. Table 1 also compares the medical schools of origin of surgical and non-surgical merit award-winners for the ten medical schools that had produced the largest numbers of award holders; the table contrasts the proportions of surgical award holders and non-surgical award holders that the alumni of each medical school achieved. Pearson's Chi-Square test showed no statistically significant difference between the distributions of the medical schools of origin for surgeon merit award-winners compared to the non-surgeon merit award-winners, *p* > 0.05. Alumni of Manchester, Cambridge, Oxford, Aberdeen, Edinburgh, Glasgow and London university medical schools accounted for 52.7% of all national merit awards held by surgeons.

**Table 1:** Top 10 medical schools; analysis by number of surgical award holders, number of non-surgical award holders and total number of award holders

Medical school	Total number of award holders	Number of surgical award holders	Percentage of surgical award holders	Number of non-surgical award holders	Percentage of non-surgical award holders
London	179	31	13.84	148	21.93
Glasgow	113	24	10.71	89	13.19
Edinburgh	84	17	7.59	67	9.93
Aberdeen	60	12	5.36	48	7.11
Oxford	45	9	4.02	36	5.33
Cambridge	43	12	5.36	31	4.59
Manchester	38	13	5.80	25	3.70
Birmingham	29	5	2.23	24	3.56
Dundee	29	6	2.68	23	3.41
Nottingham	26	7	3.13	19	2.81

Table 2 shows the effect of the size correction of medical schools on the ranking of the medical schools. London university medical schools's number one ranking before size correction dropped to a number 10 ranking after size correction. Glasgow medical school's number two ranking prior to size correction became a number one ranking after size correction.

**Table 2:** Top 10 medical school rankings by number of graduates holding merit awards; with or without size correction

Medical school	Total number of surgical award holders	Ranking by number of surgical award holders	Ranking by surgical award holders after size correction	Total number of non-surgical award holders	Ranking by number of non-surgical award holders	Ranking by non-surgical award holders after size correction
London	31	1	10	148	1	7
Glasgow	24	2	1	89	2	1
Edinburgh	17	3	2	67	3	2
Manchester	13	4	7	25	7	10
Aberdeen	12	5	4	48	4	4
Cambridge	12	6	5	31	6	6
Oxford	9	7	3	36	5	3
Nottingham	7	8	8	19	10	9
Dundee	6	9	6	23	9	5
Birmingham	5	10	9	24	8	8

Our analysis permitted comparison of the surgical A plus/platinum award holders with A/gold award holders and B/silver/bronze award holders. The surgeons with A plus or platinum awards came from only two medical schools: Manchester and Punjab. In contrast, the A or gold award holders came from 13 medical schools: Glasgow, London university medical schools, Edinburgh, Newcastle, Belfast, Oxford, Sheffield, Leicester, Manchester, Poona, Southampton, Wales and Witwatersrand. The B/silver/bronze award holders originated from 43 medical schools: London, Glasgow, Aberdeen, Edinburgh, Cambridge, Nottingham, Wales, Dundee, Ireland, Stellenbosch, Ain Shams, Cape Town, Cairo, Witwatersrand, Newcastle, Otago, Oxford, Athens, Punjab, Bombay, Tamil Nadu, Bangalore, Calcutta, Bhopal, Madras, Gujarat, Karachi, Rajiv Gandhi, Birmingham, Leeds, Bristol, Dublin, Belfast, Southampton, Liverpool, Manchester, Munich, Padua, Rome, Malta, Vienna, Washington and West Indies.

Table 3 compares the continental locations of medical schools of origin of surgical and non-surgical merit award holders for the ten medical schools with the greatest numbers of award holders. 87.1% of surgical merit award holders were from European medical schools, whereas 93.2% of the non-surgical award holders were from European medical schools. Pearson's Chi-Square test showed this to be a statistically significant difference between the continental locations of the medical schools of origin for surgeons and non-surgeon merit award holders, *p* < 0.05.

**Table 3:** A geographical comparison of the medical schools of origin of surgeon and non-surgeon merit award holders

Continental location of medical school	Non-Surgeons		Surgeons	
	Total number of non-surgeon award holders	Percentage of total number of non-surgeon award holders	Total number of surgeon award holders	Percentage of total number of surgeon award holders
Europe	629	93.19	195	87.05
Asia	27	4.00	14	6.25
Africa	9	1.33	10	4.46
North America	3	0.44	2	0.89
Australasia	6	0.89	3	1.34
South America	1	0.15	0	0
Total	675	100%	224	100%

We designated the UK and Irish medical schools as local institutions and accordingly were able to identify the international medical graduates (IMGs). 16.1% of the surgeon merit award-winners were international medical graduates, whereas 9.8% of the non-surgeon merit award-winners were IMGs. The IMGs demonstrated the greatest concentration in the B/silver/bronze category of award holders where they represented 17.6% of the surgeon merit award holders. Considering the surgeon and non-surgeon merit award-winners combined, the international medical graduates amounted to 11.4% of the total number of merit award holders.

## Discussion

### Merit awards and UK medical schools

This study is the first comprehensive peer-reviewed analysis of British merit award-winners' medical schools of origin; focusing on surgeons compared to non-surgeons. This research study serves to identify university medical schools contributing to the outcome of producing award-winning clinicians [7]. Naturally, the results of our analysis will be of importance to current and future graduates from International Medical Programs [13] in addition to local prospective medical students. This study is the first to produce a ranking of medical schools by number of merit award-winners, and so will also be of importance to medical educators.

The 2019 General Medical Council workforce study confirmed that the UK had become a significant career destination for international medical graduates [14], in fact it was stated that “For the first time, more non-UK medical graduates took up a licence to practise than UK medical graduates”. Consequently, the pool of possible medical schools of origin of the award-winners has essentially become worldwide. In our database of the 2019–20 award-winners, 85 medical schools were represented.

Our results show that after being selected using a transparent and defensible assessing and scoring arrangement [15] for merit award applicants, the majority of surgeon merit award-winners originated from a handful of medical schools. 52.7% of the surgical merit award-winners came from just seven British medical schools (Table 1). These were the London university medical schools, Glasgow, Edinburgh, Aberdeen, Oxford, Cambridge and Manchester medical schools. Moreover, there was a similar apparent concentration of merit award-winners amongst the non-surgeons, who were a natural control group for the surgical merit award holders. Here, 57.5% of the non-surgeon merit award-winners were alumni of five British medical schools: London university medical schools, Glasgow, Edinburgh, Aberdeen and Oxford. The fact that the overrepresentation of these medical schools amongst award holders applies to both surgeons and non-surgeons implies that there are common fundamental non-specialty specific factors which account for the success of these doctors.

It is recognized that students make rational decisions in the realm of education [16, 17] and information of this type is particularly relevant to a career pathway as complex as medical training that can lead to more than 20 medical specialties which then subdivide into more than 100 subspecialties. The quantitative data presented in this study provide an invaluable insight into optimum medical education pathways for students who have a sense of their likely career destinations even at an early stage in their training. Whether they are part of the increasing number of international [18] or local students, such farsighted students are likely to include graduate student entrants and mature students who wish to increase their chances of landing successfully in their chosen career. Medical school guidance will probably have valuable longevity, as recent studies have demonstrated that the differences in medical education between medical schools remain stable over the long term [19].

Irrespective of the quality of their medical training, the concentration of merit award holding doctors amongst graduates of a small number of university medical schools probably reflects additional contributions from the considerations below, either individually or in combination:

1. London university medical schools combine to form one of the largest university medical schools in Europe when assessed by number of yearly graduates. Thus, in proportion, London university is likely to be well represented in any Eurocentric merit award schemes. To investigate such an effect we performed an approximate size correction to the medical school rankings by number of award-winners, using the 2019–20 academic year undergraduate student admission numbers. Considering the surgeon merit award-winners, before the size correction London university medical schools ranked number one but fell to number ten after the correction (Table 2). A similar drop in ranking from one to seven occurred for non-surgical merit award holders from London medical schools. Clearly, a contribution to the rankings by medical school size is important but it is not clear that that school size alone can account for the concentration of award-winners in a handful of medical schools.
2. The international language of science and medicine is English and the assessment of the applications for the merit awards is performed in English. Obviously, this would tend to advantage native applicants who are graduates of UK medical schools. It could also be argued that alumni of the more traditional UK medical schools, which require a more exacting use of written English, would tend to be more successful under current merit award schemes. However, these considerations do not account for the consistent stratification of the number of award holders in UK medical schools, whether surgical or non-surgical. Furthermore, the presence of successful award holding graduates of Asian, Eastern European, South American and African medical schools suggests that language is not a major factor in preventing non-local graduates from achieving merit awards.

### Merit awards and international medical graduates

The medical schools of origin of award-winners were also analyzed by continental location, this being pertinent to the travel and relocation of medical professionals in the modern era of globalization [20]. This geographical diversity is also a good proxy for diversity of nationality amongst the merit award holders. For example, 99.4% of US medical students are American natives and 92.5% of UK medical students are UK natives (the number of international medical students that can be accepted by a medical school is capped at 7.5% by the British government). Analogously, the great majority of European medical graduates would be European natives, the great majority of Asian medical graduates would be Asian natives etc. Accordingly, the continental medical schools of origin of the surgeon and non-surgeon merit award-winners were compared (Table 3). The vast majority of surgeon and non-surgeon merit award-winners were trained in European medical schools (87.1% and 93.2%, respectively). A Chi-square test compared the continental distributions of the medical school origins of merit award holders and showed that there was a statistically significant difference between the surgeon and non-surgeon merit award-winners, *p* < 0.05. Specifically, surgical merit award holders were more likely to have trained in medical schools in Africa, Australasia, Asia or North America than their non-surgical merit award holding colleagues. A likely explanation revolves around the nature of the surgical profession itself. The greater focus on the mastery of complex manual skills that are essential to function as a good surgeon are more transferable skills than the composite range of skills that non-surgeons must acquire. It is also possible that early surgical training in these continents may allow junior doctors to gain greater experience more rapidly than in Europe. Both of these explanations could account for greater evidence of globalization that we observe with respect to the surgical merit award holders than the non-surgical merit award holders; in idealized surgical training perhaps increasing the surgical practical experience of local trainees might minimize such effects.

An important finding of this study was the greater diversity of medical school origins amongst the lowest grade of national merit award-winners than the highest grade of national merit award-winners. Specifically, the data show that all the surgeons with A + (A plus) or platinum awards originated from two medical schools representing just two continents whereas A or gold award holders came from 13 medical schools representing three continents. B, silver or bronze grade award-winners originated from 43 medical schools representing five continents. These findings appear to represent a tendency to greater globalization [20] and inclusivity effects amongst the lowest national merit awards. This is further supported by our data that show 17.6% of these surgical lower national merit award holders were international medical graduates (IMGs); in comparison with 11.0% IMGs amongst non-surgical award-winners at the same grade. The greater number of lower grade awards and the shorter time taken to attain the lower awards than the higher awards, would naturally make such demographic trends more apparent amongst the lower merit awards. Longitudinal analyses of merit award holders over the next decade would be valuable in accurately assessing whether this diversity trend progresses into the higher merit awards.

We would like to emphasize that our data, analysis and discussion above cannot quantify the presence or relative presence of discrimination for the surgical or non-surgical IMGs.

### Merit awards; undergraduate and postgraduate training of surgeons and non-surgeons

Our study is unique in directly relating merit award winners in surgery (and non-surgery) to their medical schools of origin. Accordingly, there is very little comprehensive research that relates UK medical schools to their individual performances in training medical students and the subsequent postgraduate performances of their students. Of the three studies we identified [19, 21, 22] the most comparable to ours was the MedDifs project by McManus et al*.* [19].

They studied the differences in UK medical school performances by aggregating data from 50 measures, both quantitive and qualitative, that were classified into the categories of selection of applicants, student satisfaction, curricular influences, institutional history, teaching/learning and assessment, F1 perception, foundation entry scores, postgraduate examination performance, specialty training choice and fitness to practice. Obviously and in contrast, our study was limited in not having analyzed as many educationally related factors as well as not employing a qualitative research approach. Consequently, the MedDifs study was able to compare the relationships and note both positive and negative correlations between their large number of measures (*e.g.* Problem Based Learning school graduates producing lower scores in postgraduate exams, graduates of larger medical schools tending to perform worse in their postgraduate exams and alumni of schools with greater self-regulated learning performing better in postgraduate exams). However, the MedDifs study was less able to describe the causal relationships between its measures. Both of our projects had the similar limitation of being unable to comparatively evaluate the medical schools at the level of courses within their schools.

In order to investigate the possible causalities of our presented medical school rankings for surgical and non-surgical merit award-winners, we reviewed the histories of the UK medical schools [23–32]. We noted that all 7 of the oldest medical schools, by establishment date, in the UK were present in the top 10 medical school rankings of both the surgeon and non-surgeon merit award-winners. These were all established before 1826 and comprised Birmingham (1825), Manchester (1824), Aberdeen (1786), St Bartholomew's uytryturversity (1785), Glasgow (1751), St George's London University (1733) and Edinburgh (1726). Furthermore, Oxford medical school was known to have been teaching medicine since the twelfth century and Cambridge has been teaching medicine since 1524; essentially, these two medical schools had been teaching clinical disciplines before the formal establishment process had even been created. Accordingly, with this information in mind, of the top 10 medical school rankings for surgeon and non-surgeon award-winners, 8 are the oldest medical schools in the UK.

Moreover, none of the more modern medical schools (established after 1999) are represented in our top 10 medical school merit award-winner rankings for surgeons or non-surgeons. So, Swansea (2004), Keele (2003), Hull York (2003), Brighton and Sussex (2002), Peninsula (2000), Norwich (2000) and Warwick (2000) are not represented there. Whilst it may be understandable that the younger medical schools established within the last ten years may not yet have had time to distinguish themselves to merit award levels, it is less clear that this explanation accounts for the dearth of top 10 ranked medical schools established around the year 2000.

**Fig. 1:**
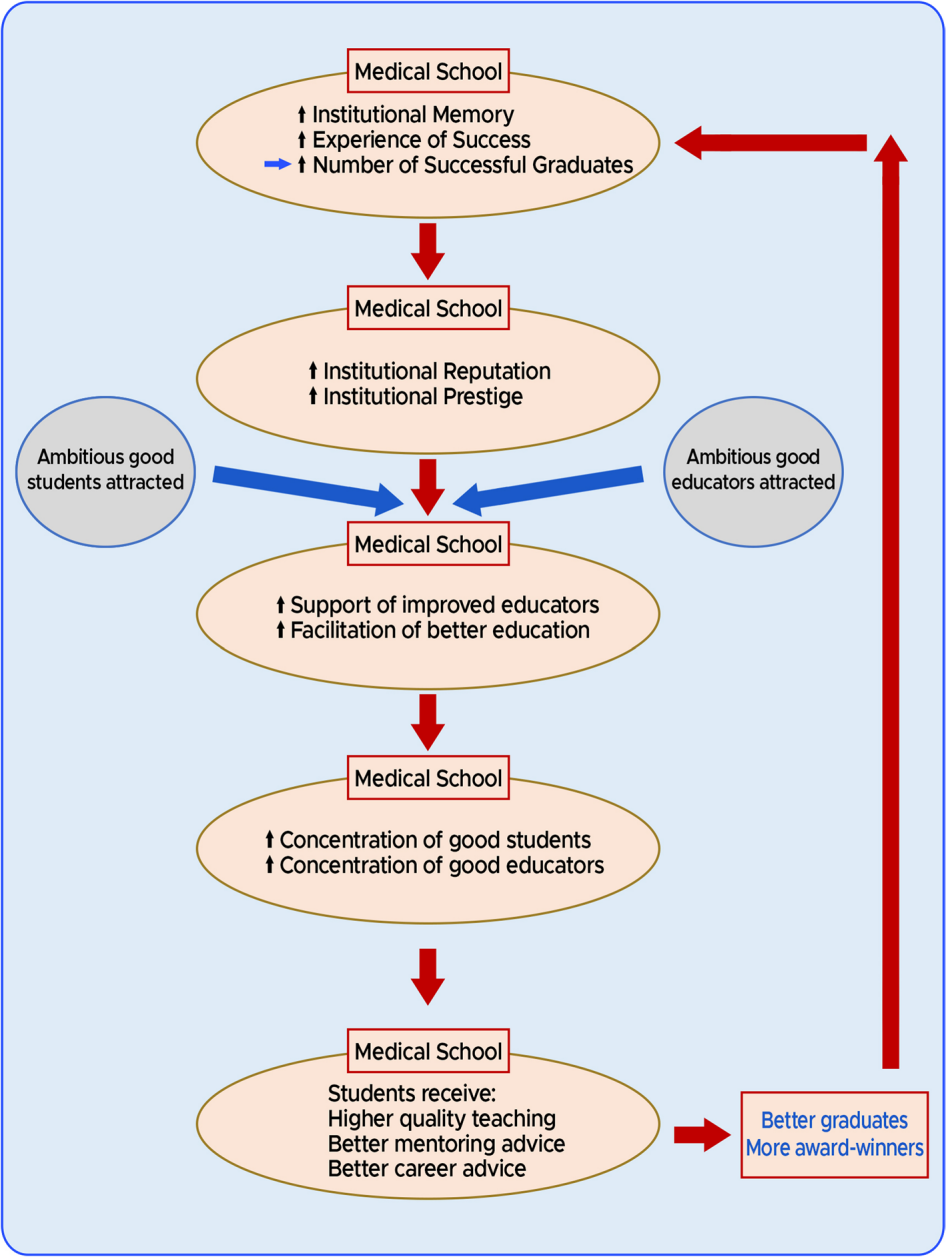
A model for creating award-winners. Cycles of institutional memory, experience and bias in education

In summary, our observations are congruous with at least a correlation between medical school age and the number of graduates becoming merit award-winners. After considering the results of our research and also accepting the previous results of the studies into UK medical school education [19, 21, 22], in Fig. 1 we propose a model describing the age dependent differential medical school performance that is consistent with the currently available data:

### Cycles of institutional memory, experience and bias in education

1. As a result of their greater longevity, the older medical schools have more institutional memory and experience in education than the younger medical schools. So, the older medical schools have a greater chance of producing successful alumni before the younger schools have even been established.
2. Because of the older medical schools apparently greater number of visibly successful alumni, they may appear more prestigious with better institutional reputations. Accordingly, ambitious and able students are more likely to be attracted to these medical schools.
3. These older medical schools with greater institutional memories and experience of producing students who achieved better postgraduate outcomes, are better placed to use this background knowledge to support and facilitate better educators and better education.
4. Therefore, these medical schools will accumulate a greater proportion of more able students and more able educators.
5. Then, the students in these university medical schools are more likely to benefit from higher quality teaching, better mentoring and better career advice.
6. Consequently, these medical schools are more likely to generate better prepared alumni who have a greater chance of becoming merit award-winners. The training of these successful doctors will add to the institutional memory and increase the medical school's successful experience in education and so the cycle will repeat
7. The action of this model will tend to reinforce stereotypes of excellence.

It should be noted that the older medical schools will necessarily have had more time to undergo more repeats of this cycle, causing a cumulative effect and thus increasing the number of successful merit award-winners originating from their schools. We also suggest that part of the reason for the differences between medical school educational performances may lie in the relative effectiveness of this cycle in different medical schools. Moreover, it should also be noted that the same studies which apply to the generation of this cycle of institutional memory and experience, also apply at the faculty/departmental levels. In the case of surgeons, a faculty or department that produces award-winning surgeons is more likely to produce more award-winning surgeons in the future. In essence, this would be a positive feedback cycle of faculty/departmental memory and experience.

It has not escaped our attention that this proposed cycle will also have positive effects on postgraduate training. For example, the award-winning and celebrated graduates of these medical schools are more likely to be perceived as leaders in surgery/medicine, as inspirational figures and to contribute to more respected postgraduate mentorship.

Finally, our model is also helpful in addressing the apparent overrepresentation of merit award-winners originating from particular medical schools—the effect of bias. As each cycle of the model occurs, greater numbers of successful graduates originating from the older medical schools accumulate in the medical community. Then these prominent graduates are more visible professionally and are also more likely to attain influential senior administrative or management positions, such as merit award allocators. As a result, implicit or explicit selection bias effects will tend to favour the graduates of these older medical schools in award allocation. Ultimately, we believe our model either wholly or partially explains the apparent concurrence of both bias and appropriate award-winning in our medical school award-winners rankings. Accordingly, it seems inevitable that the two effects of genuine appropriate award attainment and bias are linked and are likely to occur together.

In January 2022, the United Kingdom government declared that there would be an update to the Clinical Excellence Award scheme, termed the National Clinical Impact Awards, NCIA [33]. The announced objectives of this new scheme were to “(i) broaden access, (ii) make the application process simpler, fairer and more inclusive, and (iii) ensure the scheme rewards and incentivises excellence across a broader range of work and behaviours” [34]. If our cycles of institutional memory and experience model has real value, we would predict that an analysis of the future NCIA award-winners will yield similar medical school rankings to those demonstrated in our study.

## Conclusions

By using merit awards as outcome measures, our study contributes original medical education data to the pool of information that describes the demographic distribution of successful clinicians in Britain. Specifically, we identify the medical schools that are most associated with the production of award-winning surgeons. We identify the medical schools that are most associated with the production of award-winning non-surgeons. We are the first to produce a ranking of medical schools by the number of surgeon merit award-winners. We provide evidence for a rational choice of medical education centres for ambitious surgically inclined, non-surgically inclined and undecided students.

We demonstrate that international medical graduates are making substantial contributions to good surgical and non-surgical clinical practice in Britain, as judged by their concentration amongst the lower national merit award holders. We provide evidence that indicates globalization and diversity of medical school origin are being reflected in the merit awards, indicating that Britain is a credible destination for ambitious medical trainees that seek national or international success.

## Data Availability

Data from this article is available upon reasonable request to the authors. Dr Steele is the corresponding author and will make the data available. https://www.sehd.scot.nhs.uk/publications/DC20200319SACDA.pdf https://www.gov.uk/government/publications/accea-annual-report-2020 https://www.gmc-uk.org/registration-and-licensing/the-medical-register https://olr.gdc-uk.org/SearchRegister

## Abbreviation

IMG: International Medical Graduate
